# Introducing the ENHANCE (Enhancing Neurocognitive Health, Abilities, Networks, & Community Engagement) Center

**DOI:** 10.1093/geroni/igaa057.3178

**Published:** 2020-12-16

**Authors:** Walter Boot, Sara Czaja, Wendy Rogers, Neil Charness

**Affiliations:** 1 Florida State University, Tallahassee, Florida, United States; 2 Weill Cornell Medicine, New York, New York, United States; 3 University of Illinois at Urbana-Champaign, Champaign, Illinois, United States

## Abstract

Cognitive impairment (CI) refers to changes in cognition that result in difficulties remembering, learning new things, concentrating, making decisions important to everyday life, responding to environmental demands, or understanding social cues, and these difficulties can result in disability (limiting one or more major life activities). Existing and emerging technology applications hold promise for providing everyday support for older adults with CI, promoting independence and community living. However, these solutions will only be viable if they consider the needs, preferences, and abilities of older adults experiencing cognitive impairment. This talk introduces a new center, funded by the National Institute on Disability, Living, and Rehabilitation Research (NIDILRR), with the aim of supporting older adults with CI (MCI, CI as a result of traumatic brain injury or stroke) through adaptive and individualized technology solutions.

